# Evaluation of the Factor Structure of the Obstacles to Engagement Scale with Low-Income African American Parents

**DOI:** 10.3389/fped.2014.00139

**Published:** 2014-12-17

**Authors:** Deborah Winders Davis, Robert Dempster, John A. Myers, Veronnie Faye Jones, Lesa Ryan, M. Cynthia Logsdon

**Affiliations:** ^1^Department of Pediatrics, University of Louisville School of Medicine, Louisville, KY, USA; ^2^Department of Pediatric Psychology and Neuropsychology, Nationwide Children’s Hospital, Columbus, OH, USA; ^3^Department of Pediatrics, The Ohio State University College of Medicine, Columbus, OH, USA; ^4^University of Louisville School of Nursing, Louisville, KY, USA; ^5^University of Louisville Hospital, Louisville, KY, USA

**Keywords:** barriers to participation, engagement, Parents, low-income, African Americans

## Abstract

**Objective:** Parenting anticipatory guidance is one way to promote optimal child health and development and minimize disparities between children from lower socio-economic status families and their higher income peers. However, low rates of attendance at and completion of parenting programs has been demonstrated. Understanding barriers to participation has important implications. The Obstacles to Engagement Scale (OES) has been used in some populations but it has not been evaluated for use with low-income African American samples. The aim of the current study is to evaluate the factor structure of the OES with a sample of low-income, African American parents.

**Method:** Parents or legal guardians with children aged 3–8 years completed a survey in the waiting room of a primary care pediatric academic practice in an urban location in the southern United States of America (*N* = 114). Almost 87% had <12th grade education and 93% of the children received Medicaid services. The OES was one measure from a larger study and only participants with complete data on the OES were included in the exploratory factor analysis (EFA).

**Results:** The EFA did not support the previous 4-factor solution (intervention demands, personal or family stressors or obstacles, relevance of or trust in intervention, and time and scheduling demands. Instead, a 3-factor statistical solution emerged but not all items held together conceptually.

**Conclusion:** The current study supports the necessity for evaluating study instruments for use with specific populations. Larger samples are needed to disentangle the effects of educational and poverty status from race and ethnicity and to develop and validate instruments that are appropriate for the study population.

## Introduction

Early childhood is a critical period for laying the foundation for later child health, behavior, and development (1–8). The quality of parent–child interactions during early childhood has important implications for long-term health, development, and behavioral outcomes and for health and educational resource utilization (1, 9–11). Risk factors for less than optimal child development include poverty, minority status, and low levels of parent education (1, 12, 13). There are many reasons why low-income, ethnic minority families may need additional support in establishing positive parenting skills, including positive parent-child interactions, such as lack of knowledge about health and development, lower health literacy, higher levels of stress, and fewer available resources (3, 14–18). More data are needed to understand what specific factors serve as barriers for parents in receiving the support they need.

Parenting programs have been shown to be successful in improving child outcomes. With early intervention and prevention, many negative consequences can be avoided and further psychosocial dysfunction can be prevented (19). However, much remains unknown about the factors that serve as barriers for parents’ help-seeking to maximize child health and development and to prevent adverse outcomes. Parental attendance, in general, has been low for parenting programs even when their children have documented or perceived behavior problems (20–22). The Obstacle to Engagement Scale (OES) has been used in studies to understand help-seeking behavior of parents for their children’s behavior (20, 23). Data obtained while using the tool has potential to inform the development of interventions aimed at engaging parents and removing real or perceived barriers for their participation in programs that promote the development of positive parenting skills. However, care must be taken in using such instruments that have been developed and validated for other populations without validating them for other populations of interest.

Understanding barriers to parental help-seeking may lead to the development of targeted messages to increase service utilization and reduce health disparities in children who are at greatest risk such as those from low-income families and ethnic minorities. Barriers may differ with parents who differ in socioeconomic and/or educational status, race/ethnicity, age, gender, and health status, to name a few. OES (20) is a measure of potential barriers to treatment that was specifically developed to assess barriers to participation in preventative behavioral parenting classes for parents of preschool-age children. The original sample was described as economically and ethnically diverse (20) with just over half self-identifying as African American. The sample was recruited from day care centers in two metropolitan cities in the United States (*N* = 410). Others have validated the scale in samples that were predominately Caucasian (24) who were recruited from pediatric primary care settings in the Midwestern United States. To date, no studies have been found that specifically examine the psychometric properties of the scale when used with low-income African American parents. Therefore, the current study aims to determine if the factor structures previously described in other groups are the same as that found when the OES is used with a sample of low-income African American parents. Administration of the questionnaire asked parents about reasons that might prohibit their attendance at preventative parenting classes.

## Materials and Methods

### Sample

As part of a larger study, a sample of 190 parents or legal guardians completed a survey in the waiting room of a primary care pediatric academic practice that serves predominantly low-income families in an urban location in the southern United States of America. Data collection took place May through August 2011. All primary caregivers with children aged 3–8 years were invited to participate. For the larger study, parents were asked about their preferences for receiving parenting information and about issues related to stigma in addition to their obstacles to engagement. For the current study we focused on the African American participants who had complete data from the OES (*N* = 114). See Table 1 for demographic information on participants.

**Table 1:** **Demographic information of participants (*N* = 114)**.

Categorical variable	*N* = 114 (%)
Parent education
Less than high school	21 (18.6%)
Completed high school or equivalent	77 (68.1%)
More than high school	15 (13.3%)
Employment
Full-time	29 (25.9%)
Part-time	21 (18.8%)
Unemployed, looking	37 (33.0%)
Do not work due to family responsibilities	25 (22.3%)
Public resources	43 (40.2%)
Parent insurance
Medicaid/passport	71 (62.3%)
Private	10 (8.8%)
Self-pay	26 (22.8%)
Multiple insurance	7 (6.1%)
Child insurance
Medicaid/passport	106 (93.0%)
Other	8 (7.0%)
Income
<$7,355	50 (50.5%)
$7,366–14,709	16 (16.2%)
$14,210–22,065	13 (13.1%)
$22,066–29,420	10 (10.1%)
>$29,420	10 (10.1%)
One adult in family	91 (80.5%)
Single parent	93 (81.6%)
No. children in family
1	14 (12.3%)
2	35 (30.7%)
3	33 (28.9%)
4	19 (16.7%)
>4	13 (11.5%)
Continuous variables mean (SD)
Parent’s age	29.4 (6.3)
Child’s age in months	5.4 (1.7)
Other children’s age in months	7.1 (5.4)
No. adults in Family	1.2 (0.4)
No. children in Family	2.9 (1.4)

*While *n* = 114 answered at least one of the demographic questions, the sample size varied among individual questions (range: 112–114)*.

### Procedures

Parents completed a survey containing demographic characteristics of themselves and their children. Additionally, parents also completed the OES (barriers to treatment) (20). The instructions for the OES portion of the survey were as follows. “There are many reasons why parents might choose to attend or not to attend parenting classes. Listed below are some reasons that might keep parents from attending. For each item, please circle if it would stop you from attending.”

Each survey participant received an incentive of $20 upon completion. The study was approved by the University’s Institutional Review Board and conducted after receiving informed consent from the participants.

### Instruments

A researcher-developed survey was used to collect demographic information and participants also completed the OES. The OES is a 4-point Likert-type scale with responses as follows: 1 = Definitely No; 2 = Probably No; 3 = Probably Yes; and 4 = Definitely Yes. Four subscales were identified by Dumas and colleagues, which were intervention demands (four items), personal or family stressors or obstacles (four items), relevance of or trust in intervention (four items), and time and scheduling demands (two items) (20, 23).

### Statistical analysis

Exploratory factor analysis (EFA) was used to determine the factor structure of the OES in a population of low-income African American parents for comparison to the original factor structure and that previously reported in primarily Caucasian sample of parents. EFA has been conducted to validate the OES in other populations (24), and therefore, the current study extends this methodology to low-income African Americans. An EFA examines correlations among individual measure items to identify the underlying latent factors or instrument subscales. This procedure controls measurement error to identify latent factors underlying the manifest variables rather than simply condensing information provided by the manifest variables, which is typically seen in principal component analysis. The EFA used maximum likelihood estimation and oblique quartimin rotation. Factor loadings greater than 0.40 were considered significant and the squared factor loading represents the amount of variance shared between that item and the factor (or the amount of variance explained by that factor). The criterion was set to minimize the number of non-trivial cross-loadings and enhance interpretability. In addition, parallel analysis and Velicer’s minimum average partial test were used as validating procedures. If these more sophisticated techniques suggested differing factors, factors from these techniques would be reported as a replacement for the factors reported from the more traditional techniques discussed above. Fortunately, results from the traditional methods held consistent, and results from the more sophisticated techniques are not reported for continuity and to reduce possible confusion.

## Results

The EFA identified a three-factor solution on the items of the OES in a sample of low-income African Americans. The Chi-squared test demonstrated that there was not enough evidence to reject the hypothesis that the three factors were sufficient (χ^2^ = 14.32, *p* < 0.001), thus, supporting a stable factor structure. The Tucker and Lewis’ Reliability Coefficient was 0.97, exceeding the 0.94 cutoff for close fit and the root mean square error was 0.03, exceeding the 0.05 cutoff for close fit. The three factors explained 64.6% of the variance, see Table 2. The three factors’ eigenvalues were 6.19, 1.15, and 1.06, respectively (Table 2). The mean scores on the three factors were: 1.4 (SD = 0.5) for Factor 1, 1.6 (SD = 0.6) for Factor 2, and 2.0 (SD = 0.7) for Factor 3. All items, except one loaded on a factor, meaning all items were appropriate (see Table 3 for complete factor loadings). The total scale had moderate reliability (Fleiss’ Kappa = 0.64) and had good internal consistency (Cronbach’s alpha = 0.74). Cronbach’s alpha values for individual factors ranged from 0.50 to 0.87 (Table 4).

**Table 2:** **Results from the factor analysis**.

Factor	Eigenvalue	Percent of variance explained (%)	Total variance explained (%)
Factor 1	6.19	47.6	47.6
Factor 2	1.15	8.8	56.4
Factor 3	1.06	8.2	64.6

**Table 3:** **Factor loadings**.

Manifest variable	Factor 1	Factor 2	Factor 3
Would feeling frightened or nervous about being in a parenting program stop you from attending?	0.796		
Would talking about parenting with people you do not know stop you from attending?	0.522		
Would fear of being misunderstood stop you from attending?	0.453		
Would the belief that there is no hope for change stop you from attending?	0.794		
Would alcohol or drug problems in your family stop you from attending?	0.920		
Would problems with the law in your family stop you from attending?	0.909		
Would the belief that parenting programs have little connection with the problems your family is having stop you from attending?		0.651	
Would the fact that there may be too much information to learn stop you from attending?		0.524	
Would the belief that parenting programs do not work stop you from attending?		0.622	
Would lack of trust in the system or agencies stop you from attending?		0.610	
Would your work schedule stop you from attending?			0.837
Would transportation problems stop you from attending?			0.675
Would your health stop you from attending?			0.499
Would having to find time to go to meetings for several weeks in a row stop you from attending?	–	–	–

The following item did not load onto any factors: “Would finding the time to go to meetings for several weeks in a row stop you from attending?”

**Table 4:** **Cronbach’s Alpha scores**.

New Factors	Alpha Scores
Factor 1	0.868
Factor 2	0.834
Factor 3	0.502
**Original subscales**
Time and scheduling	0.700
Intervention demands	0.842
Relevance of or trust in intervention	0.860
Personal/family Stressors	0.683

## Discussion

The current study supports the necessity for evaluating the psychometric properties of study instruments for use with specific populations. Data from a sample of low-income African American parents living in an urban location in the southern United States differed from the original factor structure of a sample of adults that was economically and ethnically diverse and from a predominately Caucasian sample of parents from the Midwestern United States, which was analyzed for differences by educational group. Wilson et al. (24) found that the data from their more educated sample was similar to the original factor solution, but parents who had a high school education or less differed in their factor analysis. Similar to the current sample, they found a 3-factor solution rather than the original 4-factor solution. However, the individual items across the two samples loaded differently from each other and from the original sample (See Table 5 for a summary of all studies). Although more data are needed to further disentangle the effects of educational and poverty status from race and ethnicity, these data suggest that race and ethnicity have an effect above that of education. Larger samples with greater representation of parents who are African American, but not poor, in addition to those who are poor are needed to further understand for whom this instrument should be used.

**Table 5:** **Factors loadings for Obstacles to Engagement Scales in four separate samples**.

Items	Factor loading by study			
	Dumas et al.	Wilson et al. (College)	Wilson et al. (HS or less)	Davis et al.
Would having to find time to go to meetings for several weeks in a row stop you from attending?	1	1	1	–
Would your work schedule stop you from attending?	1	1	1	3
Would feeling frightened or nervous about being in a parenting program stop you from attending?	2	1	1	1
Would talking about parenting with people you don’t know stop you from attending?	2	–	1	1
Would fear of being misunderstood stop you from attending?	2	1	1	1
Would the fact that there may be too much information to learn stop you from attending?	2	–	2	2
Would the belief that there is no hope for change stop you from attending?	3	2	–	1
Would the belief that parenting programs have little connection with the problems your family is having stop you from attending?	3	2	1	2
Would the belief that parenting programs do not work stop you from attending?	3	2	–	2
Would lack of trust in the system or agencies stop you from attending?	3	3	1	2
Would transportation problems stop you from attending?	4	1	3	3
Would your health stop you from attending?	4	3	3	3
Would alcohol or drug problems in your family stop you from attending?	4	3	2	1
Would problems with the law in your family stop you from attending?	4	3	2	1

*The OES has four subscales or factors (20): (1) Time and scheduling (Items 1, 2), (2) Intervention demands (Items 3, 4, 5, 6), (3) Relevance of and trust in the intervention (Items 7, 8, 9, 10), (4) Personal or family stressors and obstacles (Items 11, 12, 13, 14)*.

Further instrument development research is needed. New items may need to be added or current items modified and evaluated in this population as well as other population to fully understand the barriers for help-seeking for both health promotion and prevention and for treatment of child health and development problems. As new items are developed, future studies may include both EFA as well as confirmatory factor analysis. Although the factor analysis identified a 3-factor solution, the items did not always fit together conceptually. These data clearly suggest that validation studies are needed when using instruments that were developed for use with divergent populations. Understanding the barriers for help-seeking is necessary to tailor messages and programs for the needs of various individuals and groups.

In addition to examining the psychometric properties of instruments for use in diverse populations, especially those with limited educational levels, instruments should be evaluated for reading level and cultural sensitivity. Over 90 million American adults are either functionally illiterate or have insufficient literacy skills (25). Relatedly, beyond limited general literacy skills, 14% of adults have insufficient health literacy skills and another 22% have only the very minimum adequacy (26). Literacy levels may affect the accuracy with which individuals may answer survey questions and, more importantly, low health literacy has been associated with various health behaviors and health outcomes (27–32), especially for those individuals who are most at risk for adverse outcomes such as racial/ethnic minorities. It has been shown that materials commonly used in clinical settings may not be suitable for those with limited literacy skills, especially health literacy (17, 33, 34). Health professional have begun to understand the importance of health literacy in patient and parent education and efforts are being made to develop and implement policies and practices to ensure better patient understanding of health information. Researchers must also make sure that the instruments they use in research are at the appropriate reading level and are culturally sensitive to ensure that the instruments are measuring what they intend to measure. Care must be taken with using instruments in diverse samples without evaluating the psychometric properties. Additionally, alternate methods of instrument administration such as reading the questions to the participant may be used. However, empirical data are needed to determine if oral administration is acceptable to the participant and if it improves participant understanding. In some cases, visual aids such as pictures and diagrams may ease the literacy burden for participants with limited literacy skills.

Although the data suggest that the instrument may need to be revised for use with low-income African American parents, there were some limitations to our study. The sample was a convenience sample recruited from the waiting room of a pediatric primary care office in an urban, academic, medical center in the southern United States. While the sample was representative of our clinic population, our sample and, thus, our findings, may differ from samples collected in other locations. Having only one local data collection site is a limitation. More data are needed to evaluate the usefulness of the OES in other samples. An additional limitation is that we did not have a sufficient number of participants from other races/ethnicities to do any comparisons between diverse groups nor did we have individuals who were African American with higher levels of income and/or education. Future research is needed to more fully evaluate the instrument for use across diverse groups.

In conclusion, prior to using the OES in future studies with populations that differ from the original sample, more research is needed. Additional items should be added and current items may need to be revised to reduce the literacy burden and improve cultural sensitivity. The psychometric properties should be evaluated again in another sample of low-income, African American parents. Psychometric properties should be assessed in other populations as well prior to use. Lastly, one the instrument demonstrates adequate conceptual and statistical quality; studies are needed to determine it’s usefulness in predicting appropriate outcome variables.

## Conflict of Interest Statement

The authors declare that the research was conducted in the absence of any commercial or financial relationships that could be construed as a potential conflict of interest.
